# 1-Benzoyl-3-(2,4,5-trichloro­phen­yl)thio­urea

**DOI:** 10.1107/S1600536811052780

**Published:** 2011-12-14

**Authors:** M. Khawar Rauf, Masahiro Ebihara, Amin Badshah, Imtiaz-ud-Din

**Affiliations:** a Department of Chemistry, Quaid-i-Azam University, Islamabad 45320, Pakistan; b Department of Chemistry, Faculty of Engineering, Gifu University Yanagido, Gifu 501-1193, Japan

## Abstract

The benzene and phenyl rings in the title compound, C_14_H_9_Cl_3_N_2_OS, form a dihedral angle of 40.98 (6)°. The mol­ecule exists in the thione form with typical thio­urea C—S [1.666 (2) Å] and C—O [1.227 (3) Å] bond lengths as well as shortened C—N bonds [1.345 (3) and 1.386 (2) Å]. An intra­molecular N—H⋯O hydrogen bond stabilizes the mol­ecular conformation. In the crystal, pairs of N—H⋯S hydrogen bonds link the mol­ecules into centrosymmetric dimers.

## Related literature

For information on thio­urea derivatives, see: Patil & Chedekel (1984 ▶); Baily *et al.* (1996 ▶); Namgun *et al.* (2001 ▶); Koch (2001 ▶); Wegner *et al.* (1986 ▶); Krishnamurthy *et al.* (1999 ▶); Murtaza *et al.* (2009*a* ▶,*b* ▶). For related structures, see: Khawar Rauf *et al.* (2009*a* ▶,*b* ▶). For bond-length data, see: Allen *et al.* (1987 ▶). For a description of the Cambridge Structural Database, see: Allen (2002 ▶).
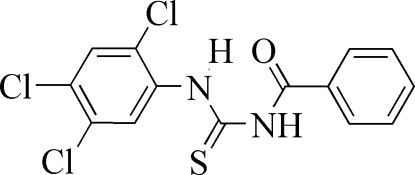


## Experimental

### 

#### Crystal data

C_14_H_9_Cl_3_N_2_OS
*M* *_r_* = 359.64
Monoclinic, 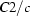

*a* = 33.111 (8) Å
*b* = 3.8413 (7) Å
*c* = 25.220 (6) Å
β = 115.995 (2)°
*V* = 2883.1 (11) Å^3^
*Z* = 8
Mo *K*α radiation
μ = 0.78 mm^−1^
*T* = 296 K
0.20 × 0.20 × 0.20 mm

#### Data collection

Rigaku/MSC Mercury CCD diffractometer
Absorption correction: multi-scan (*REQAB*; Rigaku, 1998 ▶) *T*_min_ = 0.800, *T*_max_ = 1.000
11264 measured reflections
3264 independent reflections
2686 reflections with *I* > 2σ(*I*)
*R*_int_ = 0.039

#### Refinement

*R*[*F* ^2^ > 2σ(*F* ^2^)] = 0.036
*wR*(*F* ^2^) = 0.084
*S* = 1.06
3264 reflections
190 parameters
H-atom parameters constrained
Δρ_max_ = 0.42 e Å^−3^
Δρ_min_ = −0.39 e Å^−3^

### 

Data collection: *CrystalClear* (Molecular Structure Corporation and Rigaku, 2001 ▶); cell refinement: *CrystalClear*; data reduction: *CrystalClear*; program(s) used to solve structure: *SIR97* (Altomare *et al.*, 1999 ▶); program(s) used to refine structure: *SHELXL97* (Sheldrick, 2008 ▶); molecular graphics: *ORTEPII* (Johnson, 1976 ▶) and *TEXSAN* (Molecular Structure Corporation and Rigaku, 2004 ▶); software used to prepare material for publication: *Yadokari-XG_2009* (Kabuto *et al.*, 2009 ▶).

## Supplementary Material

Crystal structure: contains datablock(s) I, global. DOI: 10.1107/S1600536811052780/bg2439sup1.cif

Structure factors: contains datablock(s) I. DOI: 10.1107/S1600536811052780/bg2439Isup2.hkl

Supplementary material file. DOI: 10.1107/S1600536811052780/bg2439Isup3.cml

Additional supplementary materials:  crystallographic information; 3D view; checkCIF report

## Figures and Tables

**Table 1:** Hydrogen-bond geometry (Å, °)

*D*—H⋯*A*	*D*—H	H⋯*A*	*D*⋯*A*	*D*—H⋯*A*
N1—H1⋯O1	0.86	1.89	2.586 (2)	137
N2—H2⋯S1^i^	0.86	2.83	3.6771 (19)	168

Symmetry code: (i) 
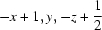
.

**Fig. 1.**
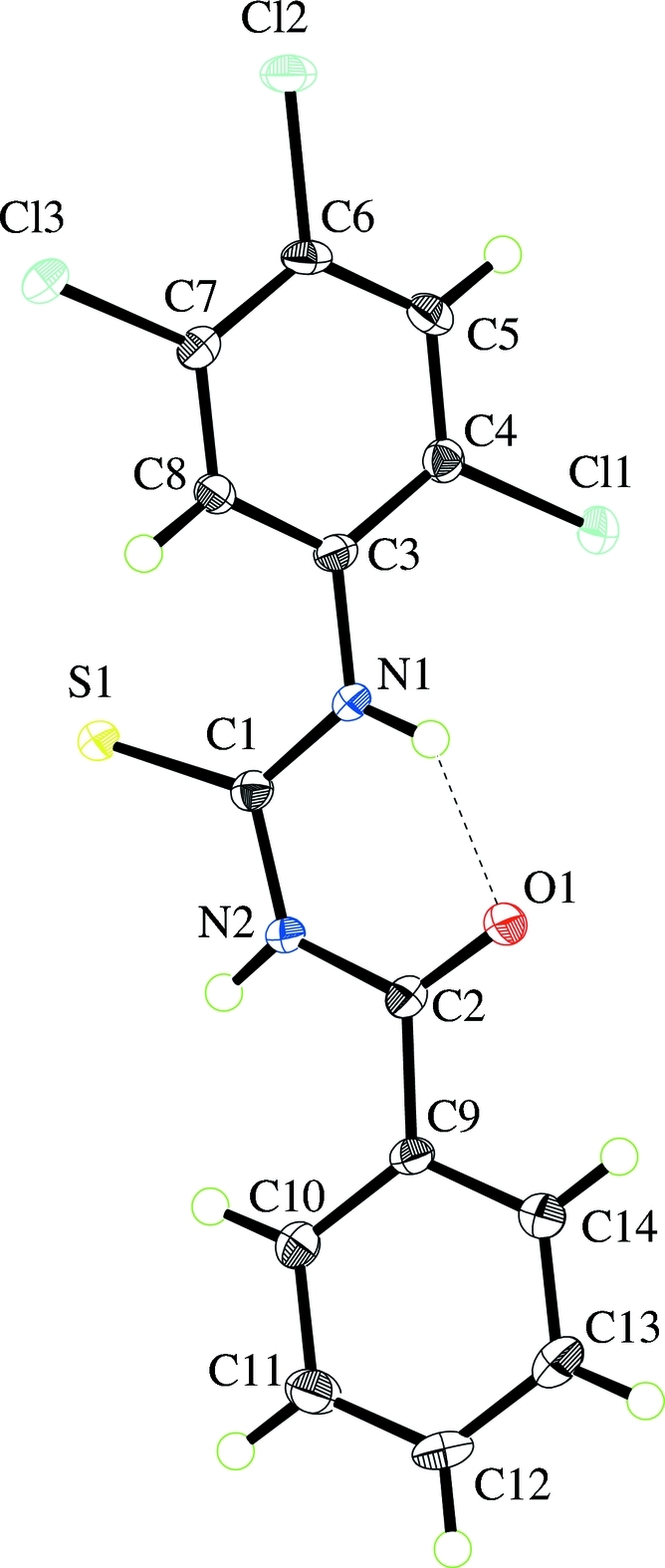
Molecular diagram of (I). Displacement ellipsoids are drawn at the 50% probability level. Hydrogen bonds shown as dashed lines.

**Fig. 2.**
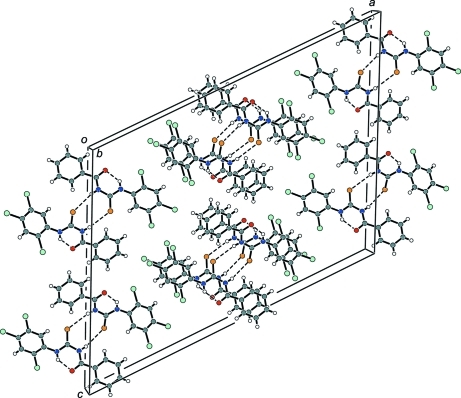
Packing diagram of (I) viewed along b-axis. Hydrogen bonds shown as dashed lines.

## supplementary crystallographic information

### Comment

Thiourea derivatives are very useful building blocks for the synthesis of a wide range of aliphatic macromolecular and heterocyclic compounds. Thus, benzothiazoles have been prepared from arylthioureas in the presence of bromine (Patil & Chedekel, 1984), 2-aminothiazoles from the condensation of thiourea with α-halocarbonyl compounds (Baily *et al.*, 1996), and 2-Methyl-aminothiazolines from *N*-(2-hydroxyethyl)-*N'*-methylthioureas (Namgun *et al.*, 2001). *N*, *N*-dialkyl-*N*-aroylthioureas have been efficiently used for the extraction of Nickle, Palladium and Platinum metals (Koch, 2001). Aliphatic and acylthioureas are well known for their antimicrobial activities (Wegner *et al.*, 1986). Symmetrical and unsymmetrical thioureas have shown antifungal activity against the plant pathogens (Krishnamurthy *et al.*, 1999). We became interested in the synthesis of these thioureas as intermediates in the synthesis of novel guanidines(Murtaza *et al.*, 2009*a* ; 2009*b*) and heterocyclic compounds for the systematic study of bioactivity and Complexation behaviour. Hence, we present here the crystal structure of the title compound, (I), Fig. 1. Comparison with *N*-benzoyl-*N'*-phenylthioureas [Cambridge Structural Database (*Mogul* Version 1.7; Allen, 2002) and (Allen *et al.*, 1987)], show the molecule to exist in the thione form with typical thiourea C—S and C—O bonds, as well as shortened C—N bond lengths. Comparison with *N*-benzoyl-*N'*-phenylthioureas (Khawar Rauf *et al.*, 2009*a*,*b*) suggests the 2,4,5-trichloro substitution on phenyl ring implies no significant effect on these bond lengths. Compound (I) (Fig. 1) shows the typical Thiourea C═S and C═O double bonds as well as shortened C—N bond lengths. The thiocarbonyl and carbonyl groups are almost coplanar, as reflected by the torsion angles C1—N2—C2—O1 [-5.0 (3)] and N1—C1—N2—C2 [1.7 (3)]. This is associated with the expected typical thiourea intramolecular N—H···O H–bond (Table 1), forming a six-membered ring commonly observed in this class of compounds (Khawar Rauf *et al.*, 2009*a*,*b*). The dihedral angles to the N1 C1 S1 N2 C2 O1 plane are 50.97 (4)° for the ring formed by C3 to C8 and 11.44 (7)° for the ring formed by C9 to C14. The crystal packing shows intramolecular N—H···O and intermolecular N—H···S H–bonds (Table 1, Fig. 2). The Cl atoms are not involved in any type of H–bonds.

### Experimental

Freshly prepared benzoylisothiocyanate (1.63 g, 10 mmol) was dissolved in acetone (50 ml) and stirred for 45 minutes. Afterwards neat 2,4,5-trichloroaniline(1.96 g, 10 mmol) was added and the resulting mixture was stirred for 1 h. The reaction mixture was then poured into acidified water and stirred well. The solid product was separated and washed with deionized water and purified by recrystallization from methanol/1,1-dichloromethane (1:1 *v/v*) to give fine crystals of the title compound (I), with an overall yield of 95%. Full spectroscopic and physical characterization will be reported elsewhere.

### Refinement

Hydrogen atoms were included in calculated positions and refined as riding on their parent atom with N—H = 0.86 Å and *U*_iso_(H) = 1.2U(N_eq_), C—H = 0.93 Å and *U*_iso_(H) = 1.2U(C_eq_).

### Crystal data

**Table:**

C_14_H_9_Cl_3_N_2_OS	*F*(000) = 1456
*M**_r_* = 359.64	*D*_x_ = 1.657 Mg m^−^^3^
Monoclinic, *C*2/*c*	Mo *K*α radiation, λ = 0.71070 Å
Hall symbol: -C 2yc	Cell parameters from 3588 reflections
*a* = 33.111 (8) Å	θ = 3.4–27.5°
*b* = 3.8413 (7) Å	µ = 0.78 mm^−^^1^
*c* = 25.220 (6) Å	*T* = 296 K
β = 115.995 (2)°	Prism, colorless
*V* = 2883.1 (11) Å^3^	0.20 × 0.20 × 0.20 mm
*Z* = 8	

### Data collection

**Table:**

Rigaku/MSC Mercury CCD diffractometer	3264 independent reflections
Radiation source: Sealed Tube	2686 reflections with *I* > 2σ(*I*)
Graphite Monochromator	*R*_int_ = 0.039
Detector resolution: 14.6306 pixels mm^-1^	θ_max_ = 27.5°, θ_min_ = 3.2°
dtprofit.ref scans	*h* = −42→31
Absorption correction: multi-scan (REQAB; Rigaku, 1998)	*k* = −3→4
*T*_min_ = 0.800, *T*_max_ = 1.000	*l* = −28→32
11264 measured reflections	

### Refinement

**Table:**

Refinement on *F*^2^	Primary atom site location: structure-invariant direct methods
Least-squares matrix: full	Secondary atom site location: difference Fourier map
*R*[*F*^2^ > 2σ(*F*^2^)] = 0.036	Hydrogen site location: inferred from neighbouring sites
*wR*(*F*^2^) = 0.084	H-atom parameters constrained
*S* = 1.06	*w* = 1/[σ^2^(*F*_o_^2^) + (0.0345*P*)^2^ + 0.4746*P*] where *P* = (*F*_o_^2^ + 2*F*_c_^2^)/3
3264 reflections	(Δ/σ)_max_ = 0.001
190 parameters	Δρ_max_ = 0.42 e Å^−^^3^
0 restraints	Δρ_min_ = −0.39 e Å^−^^3^

### Special details

**Table:**

Experimental. ????
Geometry. All e.s.d.'s (except the e.s.d. in the dihedral angle between two l.s. planes) are estimated using the full covariance matrix. The cell e.s.d.'s are taken into account individually in the estimation of e.s.d.'s in distances, angles and torsion angles; correlations between e.s.d.'s in cell parameters are only used when they are defined by crystal symmetry. An approximate (isotropic) treatment of cell e.s.d.'s is used for estimating e.s.d.'s involving l.s. planes.
Refinement. Refinement of *F*^2^ against ALL reflections. The weighted *R*-factor *wR* and goodness of fit *S* are based on *F*^2^, conventional *R*-factors *R* are based on *F*, with *F* set to zero for negative *F*^2^. The threshold expression of *F*^2^ > σ(*F*^2^) is used only for calculating *R*-factors(gt) *etc*. and is not relevant to the choice of reflections for refinement. *R*-factors based on *F*^2^ are statistically about twice as large as those based on *F*, and *R*- factors based on ALL data will be even larger.

### Fractional atomic coordinates and isotropic or equivalent isotropic displacement parameters (Å^2^)

**Table:**

	*x*	*y*	*z*	*U*_iso_*/*U*_eq_	
C1	0.43321 (7)	0.2438 (4)	0.27137 (9)	0.0156 (4)	
S1	0.435250 (18)	0.44890 (12)	0.21438 (2)	0.01721 (13)	
N1	0.39554 (6)	0.1658 (4)	0.27661 (7)	0.0169 (4)	
H1	0.3985	0.0785	0.3095	0.020*	
N2	0.47294 (6)	0.1480 (4)	0.31893 (7)	0.0161 (4)	
H2	0.4973	0.2018	0.3166	0.019*	
C2	0.47806 (7)	−0.0243 (5)	0.36979 (9)	0.0183 (4)	
O1	0.44567 (5)	−0.0919 (4)	0.37941 (7)	0.0262 (4)	
C3	0.35153 (7)	0.2166 (5)	0.23204 (9)	0.0159 (4)	
C4	0.31883 (7)	0.3736 (5)	0.24474 (9)	0.0169 (4)	
C5	0.27552 (7)	0.4225 (4)	0.20171 (10)	0.0183 (5)	
H5	0.2542	0.5274	0.2110	0.022*	
C6	0.26409 (7)	0.3141 (5)	0.14447 (9)	0.0175 (4)	
C7	0.29612 (7)	0.1512 (5)	0.13109 (9)	0.0170 (4)	
C8	0.33904 (7)	0.0996 (4)	0.17480 (9)	0.0157 (4)	
H8	0.3600	−0.0154	0.1658	0.019*	
Cl1	0.332696 (18)	0.51597 (12)	0.31608 (2)	0.02171 (14)	
Cl2	0.210441 (17)	0.39364 (12)	0.09054 (2)	0.02358 (14)	
Cl3	0.283000 (19)	0.00640 (12)	0.06075 (2)	0.02230 (14)	
C9	0.52450 (7)	−0.1244 (4)	0.41257 (9)	0.0160 (4)	
C10	0.56177 (7)	−0.1060 (5)	0.40132 (10)	0.0202 (5)	
H10	0.5589	−0.0209	0.3653	0.024*	
C11	0.60327 (7)	−0.2139 (5)	0.44354 (10)	0.0262 (5)	
H11	0.6282	−0.2027	0.4356	0.031*	
C12	0.60807 (7)	−0.3382 (5)	0.49742 (10)	0.0236 (5)	
H12	0.6361	−0.4100	0.5257	0.028*	
C13	0.57106 (8)	−0.3556 (5)	0.50921 (10)	0.0278 (5)	
H13	0.5741	−0.4375	0.5455	0.033*	
C14	0.52959 (8)	−0.2506 (5)	0.46687 (10)	0.0250 (5)	
H14	0.5047	−0.2644	0.4747	0.030*	

### Atomic displacement parameters (Å^2^)

**Table:**

	*U*^11^	*U*^22^	*U*^33^	*U*^12^	*U*^13^	*U*^23^
C1	0.0159 (11)	0.0153 (9)	0.0139 (11)	0.0006 (7)	0.0050 (9)	−0.0028 (7)
S1	0.0155 (3)	0.0206 (2)	0.0151 (3)	0.00181 (18)	0.0063 (2)	0.00292 (18)
N1	0.0143 (9)	0.0239 (8)	0.0117 (9)	0.0017 (6)	0.0048 (8)	0.0036 (7)
N2	0.0117 (9)	0.0228 (8)	0.0127 (9)	0.0001 (6)	0.0042 (8)	0.0017 (6)
C2	0.0181 (12)	0.0218 (10)	0.0136 (11)	0.0013 (8)	0.0058 (10)	0.0001 (8)
O1	0.0157 (9)	0.0441 (9)	0.0203 (9)	0.0027 (6)	0.0093 (8)	0.0103 (7)
C3	0.0135 (11)	0.0174 (9)	0.0143 (11)	−0.0006 (7)	0.0037 (9)	0.0029 (7)
C4	0.0176 (12)	0.0175 (9)	0.0162 (11)	−0.0011 (7)	0.0080 (10)	0.0005 (7)
C5	0.0143 (11)	0.0184 (9)	0.0237 (13)	0.0005 (7)	0.0096 (10)	0.0022 (8)
C6	0.0108 (11)	0.0175 (9)	0.0201 (12)	−0.0010 (7)	0.0031 (9)	0.0052 (8)
C7	0.0180 (11)	0.0170 (9)	0.0142 (11)	−0.0017 (7)	0.0054 (9)	0.0007 (7)
C8	0.0143 (11)	0.0161 (9)	0.0174 (11)	0.0009 (7)	0.0076 (9)	0.0011 (8)
Cl1	0.0205 (3)	0.0287 (3)	0.0170 (3)	0.00048 (19)	0.0092 (2)	−0.00330 (19)
Cl2	0.0140 (3)	0.0281 (3)	0.0231 (3)	0.00175 (19)	0.0030 (2)	0.0041 (2)
Cl3	0.0202 (3)	0.0295 (3)	0.0133 (3)	−0.00027 (19)	0.0038 (2)	−0.00146 (19)
C9	0.0144 (11)	0.0167 (9)	0.0138 (11)	0.0013 (7)	0.0033 (9)	−0.0008 (7)
C10	0.0217 (12)	0.0211 (10)	0.0182 (12)	0.0011 (8)	0.0091 (10)	0.0033 (8)
C11	0.0178 (12)	0.0298 (11)	0.0301 (14)	0.0022 (9)	0.0097 (11)	0.0056 (9)
C12	0.0176 (12)	0.0218 (10)	0.0213 (13)	0.0016 (8)	−0.0007 (10)	0.0033 (8)
C13	0.0291 (14)	0.0337 (12)	0.0168 (13)	0.0025 (9)	0.0066 (11)	0.0055 (9)
C14	0.0193 (13)	0.0349 (11)	0.0214 (13)	0.0032 (9)	0.0093 (11)	0.0054 (9)

### Geometric parameters (Å, °)

**Table:**

C1—N1	1.345 (3)	C6—Cl2	1.727 (2)
C1—N2	1.386 (2)	C7—C8	1.379 (3)
C1—S1	1.666 (2)	C7—Cl3	1.723 (2)
N1—C3	1.410 (2)	C8—H8	0.9300
N1—H1	0.8600	C9—C10	1.384 (3)
N2—C2	1.386 (3)	C9—C14	1.391 (3)
N2—H2	0.8600	C10—C11	1.382 (3)
C2—O1	1.227 (3)	C10—H10	0.9300
C2—C9	1.491 (3)	C11—C12	1.382 (3)
C3—C8	1.391 (3)	C11—H11	0.9300
C3—C4	1.394 (3)	C12—C13	1.383 (3)
C4—C5	1.380 (3)	C12—H12	0.9300
C4—Cl1	1.739 (2)	C13—C14	1.379 (3)
C5—C6	1.386 (3)	C13—H13	0.9300
C5—H5	0.9300	C14—H14	0.9300
C6—C7	1.394 (3)		
			
N1—C1—N2	115.16 (18)	C8—C7—C6	119.9 (2)
N1—C1—S1	125.47 (16)	C8—C7—Cl3	118.89 (16)
N2—C1—S1	119.36 (15)	C6—C7—Cl3	121.24 (17)
C1—N1—C3	124.74 (18)	C7—C8—C3	121.12 (19)
C1—N1—H1	117.6	C7—C8—H8	119.4
C3—N1—H1	117.6	C3—C8—H8	119.4
C2—N2—C1	127.76 (18)	C10—C9—C14	119.02 (19)
C2—N2—H2	116.1	C10—C9—C2	124.6 (2)
C1—N2—H2	116.1	C14—C9—C2	116.36 (19)
O1—C2—N2	121.4 (2)	C11—C10—C9	120.1 (2)
O1—C2—C9	121.06 (19)	C11—C10—H10	119.9
N2—C2—C9	117.51 (18)	C9—C10—H10	119.9
C8—C3—C4	118.10 (19)	C12—C11—C10	120.5 (2)
C8—C3—N1	121.05 (18)	C12—C11—H11	119.7
C4—C3—N1	120.81 (19)	C10—C11—H11	119.7
C5—C4—C3	121.5 (2)	C11—C12—C13	119.8 (2)
C5—C4—Cl1	118.90 (16)	C11—C12—H12	120.1
C3—C4—Cl1	119.63 (16)	C13—C12—H12	120.1
C4—C5—C6	119.57 (19)	C14—C13—C12	119.6 (2)
C4—C5—H5	120.2	C14—C13—H13	120.2
C6—C5—H5	120.2	C12—C13—H13	120.2
C5—C6—C7	119.82 (19)	C13—C14—C9	120.9 (2)
C5—C6—Cl2	118.96 (16)	C13—C14—H14	119.5
C7—C6—Cl2	121.20 (17)	C9—C14—H14	119.5
			
N2—C1—N1—C3	−175.25 (16)	C5—C6—C7—Cl3	178.95 (14)
S1—C1—N1—C3	6.3 (3)	Cl2—C6—C7—Cl3	−2.6 (2)
N1—C1—N2—C2	1.7 (3)	C6—C7—C8—C3	−1.9 (3)
S1—C1—N2—C2	−179.68 (15)	Cl3—C7—C8—C3	178.94 (14)
C1—N2—C2—O1	−5.0 (3)	C4—C3—C8—C7	2.9 (3)
C1—N2—C2—C9	175.03 (17)	N1—C3—C8—C7	−179.55 (16)
C1—N1—C3—C8	49.5 (3)	O1—C2—C9—C10	169.75 (18)
C1—N1—C3—C4	−133.0 (2)	N2—C2—C9—C10	−10.3 (3)
C8—C3—C4—C5	−1.9 (3)	O1—C2—C9—C14	−9.0 (3)
N1—C3—C4—C5	−179.45 (17)	N2—C2—C9—C14	170.96 (17)
C8—C3—C4—Cl1	178.74 (14)	C14—C9—C10—C11	0.4 (3)
N1—C3—C4—Cl1	1.2 (2)	C2—C9—C10—C11	−178.33 (18)
C3—C4—C5—C6	−0.1 (3)	C9—C10—C11—C12	−0.5 (3)
Cl1—C4—C5—C6	179.25 (14)	C10—C11—C12—C13	0.1 (3)
C4—C5—C6—C7	1.2 (3)	C11—C12—C13—C14	0.4 (3)
C4—C5—C6—Cl2	−177.30 (14)	C12—C13—C14—C9	−0.6 (3)
C5—C6—C7—C8	−0.2 (3)	C10—C9—C14—C13	0.2 (3)
Cl2—C6—C7—C8	178.26 (14)	C2—C9—C14—C13	178.98 (19)

### Hydrogen-bond geometry (Å, °)

**Table:**

*D*—H···*A*	*D*—H	H···*A*	*D*···*A*	*D*—H···*A*
N1—H1···O1	0.86	1.89	2.586 (2)	137.
N2—H2···S1^i^	0.86	2.83	3.6771 (19)	168.

Symmetry codes: (i) −*x*+1, *y*, −*z*+1/2.
